# Cytokine Registry In Stroke Patients (CRISP): Protocol of a prospective observational study

**DOI:** 10.1097/MD.0000000000020921

**Published:** 2020-07-10

**Authors:** Mudassir Farooqui, Asad Ikram, Sajid Suriya, Sidra Saleem, Syed A. Quadri, Myranda Robinson, Santiago Ortega-Gutierrez, Fares Qeadan, Enrique Leira, Surojit Paul, Atif Zafar

**Affiliations:** a Department of Neurology, University of New Mexico Health Sciences Center, University of New Mexico, Albuquerque, NM; b Department of Neurology, University of Iowa Hospitals and Clinics, Iowa City, IA; c Department of Neurology, University of Toledo, Toledo, OH; d Department of Neurology, Massachusetts General Hospital, Harvard Medical School, Boston, MA; e Department of Family and Preventive Medicine, University of Utah, Salt Lake City, UT, USA.

**Keywords:** acute ischemic brain injury, cytokine, inflammation, interleukins, protocol, stroke

## Abstract

Inflammation is an important pathophysiological process after an acute stroke (AS). Pro- and anti-inflammatory molecules (cytokines and interleukins) are the key players during this mechanism. Emerging evidence indicate that these molecules can serve as biomarkers of stroke progression and outcome and as novel therapeutics agents. The aim of this study is to explore the temporal changes in these molecules and validate them as biomarker of AS progression and neurological outcome.

The “Cytokine Registry In Stroke Patients (CRISP)” is a prospective cohort study of 600 AS patients presenting to the tertiary hospital with-in 24 h of the onset of symptoms. Plasma cytokines and interleukins will be collected at admission and 24 h after and will be measured using enzyme-linked immunosorbent assay (ELISA) to evaluate the difference in their variation among different gender, race and ethnicity and their association with various neurological outcomes. The primary exposures are biological sex (male, female) and race/ethnicity. Confounding variables include age, vascular risk factors, infarct size, stroke onset to presentation time, and identified stroke etiologies. Matched controls will be used for the comparison and evaluation of the difference among gender and race/ethnicities.

CRISP is a prospective observational study that investigates the role and relationship of molecular biomarkers identifying specific and relevant targets pertinent for monitoring the progression and outcome in AS patients.

Trial Registration: The study is registered on ClinicalTrial.gov: https://clinicaltrials.gov/ (NCT03297827).

## 1 Introduction

Stroke is a neurologic deficit occurring due to interruption of cerebral blood flow or from bleeding by a ruptured blood vessel in the brain.^[1,2]^ According to World Health Organization (WHO), it is the second leading cause of deaths worldwide.^[3]^ In the United States (US), it is the fifth leading cause of mortality killing around 140,000 Americans each year and the leading cause of morbidity and complex disability.^[4,5]^

During acute stroke (AS), the deprivation of oxygen, glucose, and other nutrients to the brain causes dysfunction of the neurovascular unit. Additionally, the breakdown of the blood–brain barrier, and induction of the inflammatory cascade results in subsequent brain damage.^[6,7]^ This is characterized by an increase in multiple pro-inflammatory and decrease in anti-inflammatory cytokines, which also correlates with progression of stroke injury.^[6,8]^ Various pro-inflammatory cytokines are observed in experimental models and in stroke patients that may correlate with infarct size and stroke outcomes.^[9–14]^ Cytokines and interleukins including TNF-α, interleukin (IL)-1, IL-6, and IL-10, have been studied as therapeutic and prognostic markers in AS.^[10,12,15,16]^ IL-10 is an anti-inflammatory molecule, observed to be associated with negative feedback, whereas IL-6 and TNF-α are reported to be increase in both ischemic and hemorrhagic stroke patients.^[11,16,17]^ Moreover, glial cell markers such as S100B and various other cytokines have also been reported to be associated with infarct size, neurological outcome, and prognosis among these patients.^[10,17–21]^

The inflammatory mechanism and the interaction between the pro- and anti-inflammatory cytokines in AS patients is still ambiguous. Although, studies have indicated changes in systemic cytokine profile in response to experimental stroke, few studies have assessed peripheral cytokine levels in human stroke patients and their association with the neurological outcome.^[10]^ As such, for the use of cytokines as potential biomarkers and prognosis of stroke, it is important to design clinical stroke studies on peripheral cytokine expression that focuses on the temporal profile, comorbidities, and demography.

## 2 Trial objective

The primary objective of the *“*Cytokine Registry In Stroke Patients (CRISP)” is to evaluate

1. the plasma concentration and time course of these pro- and anti-inflammatory cytokines and interleukins
2. identification and establishment of a blood base bio-marker that can corroborate with the neurological outcome in these AS patients.

Our primary hypothesis is that temporal change in any one or more cytokine or interleukin levels during this early critical phase could be an important predictor of stroke severity. Moreover, separate sub-studies will explore and compare plasma cytokine levels based on gender, race, and ethnicity.

## 3 Methods

### 3.1 Study design

**Figure 1:**
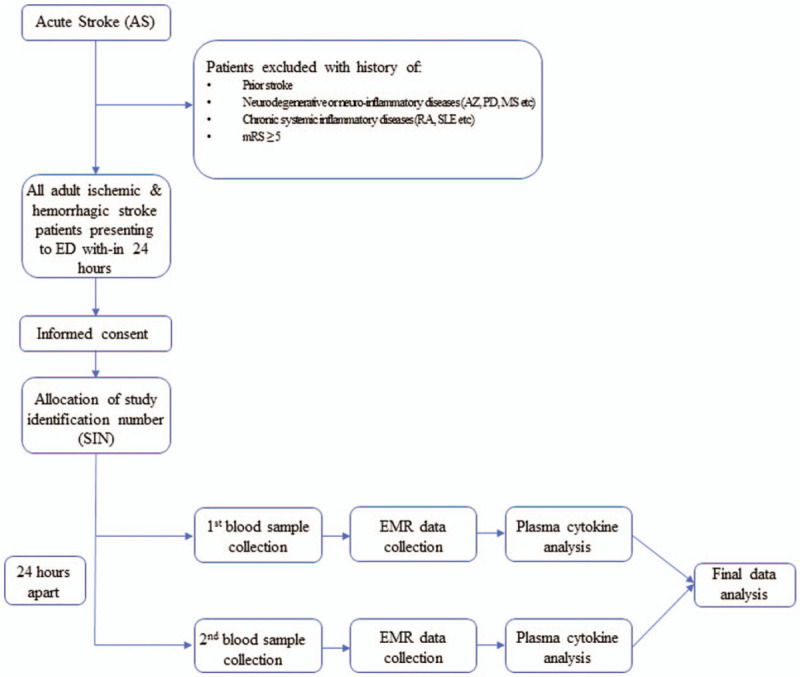
Schematic representation of the CRISP study trial.

This is a prospective patient cohort registry (NCT03297827). Six hundred patients with the diagnosis of AS (IS and HS) will be recruited over the period of 3 years (2018–2021). The overview of the study design is presented in Figure 1. Plasma samples will be collected on admission and after 24 h. Plasma ILs (1, 4, 6, 10, 17, 23, 33, 36, and 37), platelet-derived growth factor (PDGF), vascular endothelial growth factor (VEGFM), tumor necrosis factor (TNF-a), ANNULIN, matrix metallopeptidase (MMP) 9 and 12, nuclear factor kappa-light-chain-enhancer of activated B cells **(**NFk-B), myeloperoxidase (MPO), glia-maturation factor (GMF), SI00 calcium-binding protein B (S100B), and GM6001 will be measured using enzyme-linked immunosorbent assay (ELISA).

### 3.2 Eligibility criteria

Inclusion criteria:

1. All stroke patients (hemorrhagic and ischemic) presenting within 24 h of onset based on clinical and radiographic examination
2. Adult male/female patients ages >18 years old

Exclusion Criteria:

1. History of prior stroke or any other neurodegenerative or neuroinflammatory disease (Dementia of Alzheimer's type, Parkinson's disease, multiple sclerosis, etc) and acute infectious diseases (respiratory tract infections, urinary tract infections, etc).
2. Patients with chronic inflammatory systemic illness (including premorbid diagnosis of rheumatoid arthritis, systemic lupus erythematosus, sarcoidosis or other immune-mediated diseases)
3. Individuals ages <18 years
4. Pregnant women
5. Prisoners
6. Low likelihood of survival >24 h, based on clinical assessment (mRS ≥ 5)

### 3.3 Data Collection

Patients who will meet all inclusion criteria/exclusion criteria will be consented. Two sets of plasma samples will be obtained from individual participants; one upon admission to the hospital, and the second sample 24 h apart from the first one. Both sets of blood samples (3–5 mL) will be collected from peripheral veins according to the routine puncture method as standard of care. After centrifugation, the resulting plasma supernatants will be stored at −80°C. The samples will be transported and handled according to the federal guidelines. Additional hospital data will be collected by chart review. This includes last known well or the time of onset of symptoms (according to patient or bystander), the presence of any neurological deficit or hemiparesis (if applicable), demographics, vital functions, neurological examination (NIHSS), imaging include non-contrast CT scan, CT angiography (CTA), digital subtraction angiography (DSA), and/or CT perfusion, clinical data including medical history, comorbidities, medication use, laboratory results, and physical examination will be collected using the chart review. Information regarding the corresponding treatment including door to needle time and door to groin time will be also collected. Other parameters like length of hospital stay, discharge location, and modifies Rankin Scale (mRS) at 3 months will be collected as part of the protocol. All paper consents will be secured in the locked cabinet in the principal investigator (PI) office. The PI's research team will be responsible for consenting, collecting samples, storage in secure refrigerator, running the assays and entering the inferences and other clinical details into a secure REDCap database.^[22]^ REDCap (Research Electronic Data Capture) is a secure, web-based application designed to support data capture for research studies, providing

1. an intuitive interface for validated data entry;
2. audit trails for tracking data manipulation and export procedures;
3. automated export procedures for seamless data downloads to common statistical packages; and
4. procedures for importing data from external sources.

### 3.4 Study outcomes

The primary objective of this study is to measure the levels of the reported cytokines and other analytes that have a continuous measure, from the plasma of AS patients using standardized ELISA kits. The primary exposures are biological sex (male, female) and race/ethnicity (American Indian/Alaskan Native, Asian/Pacific Islander, African American, Whites, and Hispanics). Confounding variables to adjust for are age, vascular risk factors, medications (anti-inflammatory), infarct size, stroke onset to presentation time, and identified stroke etiologies (based on TOAST criteria).

### 3.5 Recruitment

This study will be performed at the tertiary care hospital. It will be conducted in accordance with the principles of Good Clinical Practice (GCP). The study protocol was approved by the Institutional Review Board (IRB). Study participants will be recruited through the department of Neurology after the written informed consent. Informed consent can be very challenging in AS patients. Patients may suffer from language deficit and/or other cognitive impairment that can hamper the informed consent procedure. The primary stroke team and/or the physician provider will assess the mental competence of the potential study participant to determine whether they are capable of giving informed consent. Hence, if the patient is ineligible the study will include the legal authorized representative (LAR) as part of the informed consent process. A study team member will obtain both verbal and written consent and will explain the study protocol to the potential participants. All the patients with the diagnosis of AS will be approached for the consent procedure. In addition, a flyer detailing an overview of the study protocol, as well as a copy of the formal consent form, will be given to potential trial participants. After obtaining the informed consent, the study team member will assign a unique study identification number (SIN) and enter the information in the REDCap database. After informed consent, a pre-randomization assessment for every recruiting patient will be done, evaluating their medical records for any autoimmune and/or inflammatory disorders.

### 3.6 Data analysis and sample size

The study team along with the core statistical division will evaluate all the study trial outcomes. One Way Analysis of Covariance (ANCOVA) will be employed, followed by post hoc tests while adjusting for multiple comparisons using the Sidak adjustment. As a secondary analysis, we will also look at the correlation network of these cytokines after stratification by sex and race/ethnicity. According to the ANCOVA model, based on 80% power and 5% significance level, the minimum sample size is 420 AS participants. The primary exposures are biological sex (male, female) and race/ethicity (American Indian/Alaskan Native, Asian/Pacific Islander, African American, Whites, and Hispanics). Confounding variables adjusted in the model include age, vascular risk factors, infarct size, and identified stroke etiologies. Matched controls will be used for the comparison and evaluation of the difference among age, gender, and race/ethnicities.

### 3.7 Data management

All the collected data for the CRISP study trial will be entered into REDCap database. Restricted entry will be provided only to the study team members. Missing data or specific errors will be detected and summarized along with detailed descriptions during the bi-weekly reports presented to the PI. The PI will check the original case reporting forms (CRF's) to determine any inconsistency and respond to the data reports generated. Data access will be restricted via password protection to only those individuals who are authorized to work on the study trial. Specific privileges within the database will be employed to limit the types of data that authorized users may access to the minimum required by their role in the study trial. Participant files will be stored in numerical order in a secure place. Original study forms will also be kept in an accessible location for the team in the research office. These files will be maintained in storage for a period of at least 5 years after study completion.

### 3.8 Data monitoring and quality assurance

An independent Data Safety Monitoring Board (DSMB) will oversee the CRISP trial. The DSMB is comprised of independent, multidisciplinary experts reviewing the general conduct of the trial, and providing recommendations for the continuation, modification or termination of the trial. The DSMB will examine the data to assure the regulatory bodies, the public and the National Institute of Health that conflicts of interest do not compromise either patient safety or trial integrity. The DSMB will convene before study initiation and for annual reviews. All unexpected adverse events related to the trial will be recorded in the trial database and reported to the DSMB and Institutional Review Board (IRB).

#### 3.8.1 Confidentiality of data

All the data will be stored on a secure and encrypted password-protected network database, in compliance with the hospital approved information technology security. All the patients will be assigned a unique SIN. Only the PI and the sub-investigators can access the key linking SIN and the patient's personal identifiers. The paper record for the patient's clinical data will be stored in a locked cabinet located in the PI's and/or research team office. After the completion, the data will be stored for 5 years.

## 4 Dissemination

The study PI along with the sub-investigators will be responsible for all the major decisions regarding changes to the protocol and will communicate these changes to the IRB. All the data and demographic information including the SIN with the identifiers will be accessed only by the PI and the sub-investigators and as necessary for completion of trial follow-up tasks. The PI will have access to all the data.

The main study trial results will be disseminated via peer-reviewed publication in an international journal. The results will also be presented at local, national and international conferences on stroke and neurology. Moreover, the results from the secondary analyses will be also presented in the conferences and in the form of manuscript for the publication in peer-review journal. Dissemination of the results to the study trial participants and their family members will be available on request. Additionally, regular updates of the study trials will be available to the public on ClinicalTrials.gov.

## 5 Discussion

The study will prospectively create a comprehensive database that will include demographics emphasizing patient ethnicities, clinical symptoms, vascular comorbidities, findings on carotid, and cardiac ultrasounds, vascular and brain imaging along with the plasma levels of cytokines and neural markers. This will potentially enable scientists and clinicians to identify specific and relevant molecular targets pertinent to human beings.

The results of the study will help to better understand stroke molecular pathophysiology with emphasis on ethnic and biological sex variations. Furthermore, role of newer interleukins like IL-33 and IL-37 in inflammatory disease and cardiovascular diseases is now being acknowledged.^[23,24]^ However, there is a lack of literature explaining the interaction of these newer interleukins in stroke. The present study will help delineate the role of these newer interleukins and other novel cytokines in AS patients while considering ethnic and gender related genetic predisposition.

In conclusion, CRISP is a prospective observational study that investigates the role and relationship of molecular biomarkers identifying specific and relevant targets pertinent for monitoring the progression and outcome in AS patients.

## Author contributions

Authorship will be given to the key personnel involved in the trial.

AZ, MF, SAQ, EL, SP, FQ, SOG were involved with the conceptualizing of the trial design, AI, SS, SS, MR are involved with the patient safety protocols, recruitment and data monitoring. All authors agree to be accountable for the accuracy of the trial.

**Conceptualization**: Atif Zafar, Mudassir Farooqui, Enrique leira, Santiago Ortega-Gutierrez, Syed Quadri, Surojit Paul.

**Data curation**: Myranda Robinson, Asad Ikram, Sajid Suriya.

**Formal analysis**: Fares Qeadan.

**Funding acquisition**: Fares Qeadan, Surojit Paul, Atif Zafar.

**Investigation**: Mudassir Farooqui, Asad Ikram, Sajid Suriya, Sidra Saleem, Myranda Robinson, Fares Qeadan, Surojit Paul,AtifZafar.

**Methodology**: Mudassir Farooqui, Asad Ikram, Sajid Suriya, FaresQeadan, Surojit Paul, Atif Zafar.

**Project administration**: Mudassir Farooqui, Sajid Suriya, Surojit Paul,Atif Zafar.

**Resources**: Atif Zafar.

**Supervision**: Surojit Paul, Atif Zafar.

**Validation**: Atif Zafar.

**Visualization**: Atif Zafar.

**Writing – original draft**: Mudassir Farooqui, Sajid Suriya, Sidra Saleem, Atif Zafar.

**Writing – review & editing**: Mudassir Farooqui, Sajid Suriya, Syed Quadri, Santiago Ortega-Gutierrez, Fares Qeadan, Enrique Leira, Surojit Paul, Atif Zafar.
